# Potential of Polyamide Nanofibers With Natamycin, Rosemary Extract, and Green Tea Extract in Active Food Packaging Development: Interactions With Food Pathogens and Assessment of Microbial Risks Elimination

**DOI:** 10.3389/fmicb.2022.857423

**Published:** 2022-03-15

**Authors:** Simona Lencova, Hana Stiborova, Marcela Munzarova, Katerina Demnerova, Kamila Zdenkova

**Affiliations:** ^1^Department of Biochemistry and Microbiology, University of Chemistry and Technology, Prague, Czechia; ^2^NanoMedical, Liberec, Czechia

**Keywords:** food packaging, food microbiology, polyamide, nanofibers, natamycin, rosemary, green tea

## Abstract

Increasing microbial safety and prolonging the shelf life of products is one of the major challenges in the food industry. Active food packaging made from nanofibrous materials enhanced with antimicrobial substances is considered a promising way. In this study, electrospun polyamide (PA) nanofibrous materials functionalized with 2.0 wt% natamycin (NAT), rosemary extract (RE), and green tea extract (GTE), respectively, were prepared as active packaging and tested for the food pathogens *Escherichia coli*, *Listeria monocytogenes*, *Salmonella enterica*, and *Staphylococcus aureus*. The PAs exhibited: (i) complete retention of bacterial cells reaching 6.0–6.4 log_10_removal, (ii) antimicrobial activity with 1.6–3.0 log_10_suppression, and (iii) antibiofilm activity with 1.7–3.0 log_10_suppression. The PAs prolonged the shelf life of chicken breast; up to 1.9 log_10_(CFU/g) suppression of total viable colonies and 2.1 log_10_(CFU/g) suppression of *L. monocytogenes* were observed after 7 days of storage at 7°C. A beneficial effect on pH and sensory quality was verified. The results confirm microbiological safety and benefits of PA/NAT, PA/RE, and PA/GTE and their potential in developing functional and ecological packaging.

## Introduction

Nowadays, great emphasis is placed on the quality, freshness, and safety of food. Indisputably, food packaging plays the main role in food protection. Standard food packaging, without any additional functionality, is usually made from polymers, such as polyethylene, polypropylene, polystyrene, or polyethylene terephthalate (Duncan, 2011). But new food packaging technologies are developing in response to consumer demand for minimally processed food. Over the last few decades, active food packaging from nanofibrous materials has become one of the most innovative approaches. Such packaging has active functions that go well beyond providing an inert passive barrier and protecting food from outside contamination by light, gases, dusts, odors, water, and microorganisms. Nanofibers with their unique properties, such as air permeability and at the same time the retention of unwanted elements from the external environment, may be used as suitable packaging. Their air permeability can be affected and controlled primarily by the fabric weight and further by the morphology of nanofibers, specifically with surface density and fiber diameter (Abuzade et al., 2012). Through interaction with the food product itself, nanofibrous active packages can extend shelf life without the need for preservatives and can prevent the spread of foodborne pathogens (Labuza and Breene, 1989).

Various polymers are used for functional packaging development; current research focuses on sustainable and economical materials, such as wood-based polymers (cellulose, hemicellulose and starch) and protein-based polymers (keratin, soy protein, and gelatin; Asgher et al., 2020). In addition to the mentioned biomaterials, synthetic polymer polyamide (PA) provides the mentioned advantages and is considered one of the suitable alternatives for packaging materials development (Borzi et al., 2019; Tyuftin and Kerry, 2020), especially in connection with nanotechnologies. PA nanofibers prepared *via* electrospinning can be used for food packaging due to their suitable mechanical properties, such as mechanical resistance, strength under both dry and wet conditions, stability against chemical agents, flexibility, and excellent retention ability (Lencova et al., 2021b). At the same time, contrary to the biomaterials, PA is not degradable, can be used repeatedly, and thus provides environmental benefits (Winnacker, 2017). Despite such advantageous properties, PA itself is not antimicrobial and is not able to prolong foods shelf life by the prevention of microbial contamination (Lencova et al., 2021a,b).

In fact, infections and intoxications resulting from the ingestion of foods contaminated with bacteria or bacterial toxins are currently the most common type of illness worldwide and can lead to very serious health issues. The bacteria *Escherichia coli*, *Listeria monocytogenes*, *Salmonella enterica*, and *Staphylococcus aureus* are among the most common food pathogens causing alimentary infections and intoxications in developed countries (Galié et al., 2018). Pathogenic strains of *E. coli* contaminate both food (drinking water, fruits, vegetables, meat, etc.) and food manufactories (Galié et al., 2018); and usually cause diarrhea or infectious diseases (Yang et al., 2017). *Listeria monocytogenes* contaminates, for example, dairy products, meat, seafood, causes gastroenteritis, in immunocompromised persons, it may cause life-threatening disease, listeriosis, and in pregnant women, it may cause spontaneous abortion (Galié et al., 2018). Poultry meat is a typical reservoir of *S. enterica*, which causes gastroenteritis and septicemia (Galié et al., 2018). *Staphylococcus aureus* mainly contaminates meat products; produces staphylococcal enterotoxins responsible for diarrhea, vomiting, and acute toxic shock (Alibayov et al., 2015). A common feature of these bacteria is the ability to form biofilms, complex accumulations of microbial cells (Rodney, 2002) in which they are more resistant to adverse effects. Biofilms of food pathogens on food matrixes, food packaging, and food factory equipment, are one of main causes of foodborne disease development (Galié et al., 2018).

With the need for producers to strictly control the microbiological quality of their products, active antimicrobial food packaging can be very beneficial. Antimicrobial packaging can reduce, inhibit, or slow down the growth and biofilm formation of food pathogens that may be present in the packed food or the packaging material itself. In this way, they can help to prevent the spread of foodborne disease. Basic principles and types of antimicrobial packaging involve: (i) the addition of pads containing antimicrobial reagents into packages, (ii) the incorporation of antimicrobial agents directly into polymers, (iii) the coating of polymer surfaces with antimicrobial agents, (iv) the immobilization of antimicrobial agents to polymers by covalent or ion linkages, and (v) the use of polymers that are antimicrobial themselves without the need for any functionalization (Appendini and Hotchkiss, 2002). The second of the above approaches is currently preferred by far.

The food-protective effect of materials which are not antimicrobial themselves can be enhanced by the addition of various antibacterial substances, such as antibiotics, nanoparticles (NPs), or natural extracts. Antibiotics are not preferred due to their possible side effects, such as the development of antibiotic resistance. NPs, mainly Ag NPs, are widely studied as potential antimicrobials for food packaging (Carbone et al., 2016; Istiqola and Syafiuddin, 2020). However, they are not considered safe for food packaging due to the risks associated with their potential migration into food and probable cytotoxicity and genotoxicity (Carbone et al., 2016; Istiqola and Syafiuddin, 2020). Natural substances are therefore the most acceptable antimicrobial agents.

A wide range of natural antimicrobial substances, for example, natamycin (NAT; Duran et al., 2016), rosemary extract (RE; Piñeros-Hernandez et al., 2017), or green tea extract (GTE; Wrona et al., 2017), have great potential for application in active food packaging. Some of them have already been tested for this purpose and provided promising results. They are considered safe for human health due to their natural origin and current trends favor the use of natural substances instead of synthetic preservatives (Galié et al., 2018). However, food packaging (nano)materials functionalized with the above-listed substances are not common.

Until now, a variety of promising active food packaging from nanofibers functionalized with other substances was developed (Duan et al., 2021; Göksen et al., 2021). In terms of microbiological safety and better benefits of these materials, usually only antimicrobial activity on model organisms or food samples is monitored (Veras et al., 2020; Göksen et al., 2021), whereas their ability to suppress biofilm formation and retain microbial cells is studied rarely. According to our knowledge, only one study focusing on all the mentioned aspects—antimicrobial, antibiofilm, and microbial barrier properties—of materials potentially applicable as food packaging has been published, but the tests were not further performed on food samples (Babu et al., 2016).

In this study, we prepared PA nanofibrous materials functionalized with NAT, RE, and GTE, respectively, which could be potentially used for the development of active food packaging. We hypothesize that functionalized PAs will reduce the overall risks associated with microbial food contamination. To prove this hypothesis, we tested: (i) the retention ability of the PAs for bacterial cells, (ii) the antibacterial properties of NAT, RE, and GTE and all functionalized PAs, (iii) the ability to suppress biofilm formation and, (iv) the prolonged shelf life of chicken breast with non-inoculated and inoculated meat with *L. monocytogenes* CCM 7202. The bacterial strains *E. coli* CCM 4517, *L. monocytogenes* CCM 7202, *S. enterica* ssp. *enterica* serovar Enteritidis CCM 7189, and *S. aureus* CCM 3953 were selected for the analysis as representatives of the most important foodborne bacterial pathogens (Galié et al., 2018). This study is the first one focusing specifically on the overall microbiological analysis of the nanomaterials potentially applicable as food packaging/food packaging component on model microorganisms as well as on food samples. It is one of the essential steps of functional packaging development, which have been totally overshadowed by the emphasis on physical and mechanical properties evaluation—other essential part of packaging development—so far.

## Materials and Methods

### Bacterial Strains and Culture Conditions

Gram-positive bacterial strains *L. monocytogenes* CCM 7202 (eq. ATCC 13932) and *S. aureus* CCM 3953 (eq. ATCC 25923) and Gram-negative bacterial strains *E. coli* CCM 4517 (eq. ATCC 8739) and *S. enterica* ssp. *enterica* serovar Enteritidis CCM 7189 (eq. ATCC 13076) were obtained from the Czech collection of microorganisms (CCM, Czechia). Bacterial suspensions were prepared from pure bacterial cultures grown in Tryptone Soya Broth (TSB, Oxoid, Great Britain) and used for further studies.

### Antimicrobial Substances

Natamax® (natural antimycotic NAT blended with lactose), Guardian™ Rosemary extract 09 (natural RE and food-grade carrier system), and Guardian™ Green tea extract 20 M (natural GTE with maltodextrin as carrier) were purchased from the manufacturer, Danisco A/S (Denmark). The information contained in this publication is based on our own research and development work and is to the best of our knowledge reliable. Users should, however, conduct their own tests to determine the suitability of the products for their work.

### Minimal Inhibitory Concentration and Minimum Inhibitory Concentration for Biofilm Formation

#### Disc Diffusion Method: MIC_dd_ Determination

The inoculum of the tested bacteria was adjusted to optical density (OD) 0.5 McF in Mueller–Hinton Broth (MHB, Oxoid, Great Britain); 100 μl of the inoculum was spread onto Mueller–Hinton agar (MHA, Oxoid, Great Britain). Six-mm-diameter antibiotic disks (Macherey-Nagel, Germany) were impregnated (10 min, room temperature) with various concentrations (10.0–0.16 wt%) of NAT, RE, and GTE, respectively. The disks were placed onto inoculated MHA and incubated (37°C, 24 h). The inhibition zones formed around the disks were measured. The sensitivity of the tested bacterial strains to NAT, RE, and GTE, and MIC_dd_ (expressing the lowest concentration of the substance which formed a clear inhibition zone around the disc) was determined. Tests were performed in technical and biological triplicates.

#### Microdilution Method: MIC_md_ Determination

The inoculum of the tested bacteria was adjusted to OD 0.5 McF in MHB; 100 μl of the inoculum was added to the sterile 96-well microtiter plate (Gama group, Czechia) and mixed with 100 μl of NAT, GTE, or RE, respectively, at the required concentration. Active substances were serially diluted in the final concentrations range 10.0–0.04 wt%. The inoculum served as a positive control of bacterial growth; sterile MHB served as a control of sterility. The absorbance at 620 nm was measured spectrophotometrically (Tecan, Switzerland) both before and after the cultivation (24 h, 37°C). The difference in measured data was considered as the indicator of bacterial cell viability in the presence of the tested substances and was used for the MIC_md_ determination. MIC_md_ was the lowest concentration of the substance, which suppressed bacterial growth completely; A_620nm_ < 0.01 was considered to be total inhibition. Tests were performed in technical and biological triplicates.

#### Microdilution Method: MIC_BF_ Determination

Subsequently, the biofilm formed in the wells was quantified using CV staining. After MIC_md_ measurement, the wells were washed with 200 μl of sterile distilled water (five times) and dried at room temperature (45 min). One hundred fifty microliter of 0.1% crystal violet (CV, Sigma-Aldrich, United States) was added to the wells; after staining (45 min, room temperature), the wells were washed with 200 μl of sterile distilled water (five times). For CV stain elution, 200 μl of 96% ethanol was added; after elution (15 min, room temperature), 100 μl of the solution was transferred into a sterile microtiter plate, and biofilm formation was measured spectrophotometrically at 595 nm. After background subtraction, MIC_BF_s were determined. MIC_BF_ expressed the lowest concentration of the substance which suppressed bacterial biofilm formation completely; A_595nm_ < 0.09 was considered to be total inhibition. Tests were performed in technical and biological triplicates.

### Nanomaterial Preparation

The presented PA nanofibrous materials were prepared by a needleless electrospinning method using a Nanospider™ NS 1WS500U (Elmarco, Czechia) with a one spinning electrode wide 500 mm. Nanofibers were electrospun from polymer PA6 [Ultramid B24 N 03, BASF, Germany; an average molecular weight (M_w_) of 55,600 g/mol] solutions in a solvent containing acetic acid (Penta, Czechia; M_w_ of 60.05 g/mol) and formic acid (Penta, Czechia; M_w_ of 46.03) in a 2:1 ratio (v/v). The PA6 concentration in the solution was from 12% to 14% (w/w), which was determined in our previous research as optimal for the production of PAs with required morphology (Munzarová et al., 2020). The solution was dissolved by stirring for 24 h at 65°C before spinning. For electrospinning, a driving voltage of 90 kV was applied. Electrospinning was performed at 20°C–22°C and relative humidity 40%. The forming fibers were collected on polypropylene antistatic non-woven backing material placed 240 mm above the active electrode and rolled at a speed of 85 mm/min. Electrospinning of the NAT, RE, or GTE supplemented nanofibers was carried out under the same conditions. The solutions for spinning were prepared by adding 2.0% (w/w) of NAT, RE, or GTE, respectively, to the prepared 12% (w/w) PA6 solution (blending electrospinning). The preparation of functionalized PAs follows up on our previous research, in which stability, reproducibility, and homogeneity of nanomaterials were verified (Munzarová et al., 2020).

### Nanomaterial Characterization

The prepared PA nanofibrous materials were characterized in terms of their morphology. Scanning electron microscopy (SEM) using a Tescan Vega3 SB Easy Probe (TESCAN, Czechia) was used to assess PA homogeneity and to investigate the possible effect of the functionalization on PA morphology. Before SEM analysis, the PA nanofibers were sputter-coated with gold (14 nm). The software NIS Elements (Nikon, Japan) was used to perform the morphological analysis. Fiber diameter, surface density, and air permeability of the PAs were determined. Fiber diameter was evaluated from 100 measurements of different areas (Lencova et al., 2021a), surface density (the thickness of PAs) was measured using a Corp ID-C112XB (Mitutoyo, Czechia), and air permeability was measured with a TEXTEST FX 3300 (TexTest Instruments, Switzerland). The air permeability measurement was done with standardized pressure gradient at room temperature; was based on measuring the amount of air that passed between the opposite sides of the PA, relative to the surface area and time. The air flow pressure was 200 Pa.

### Permeability Assay for PA Nanomaterials

The permeability assay was performed as described in Lencova et al. (2021b). Briefly, single-bacterial suspensions (3 ml, OD 1 McF) were filtered through a sterile (UV radiation, room temperature, 20 min) PA membrane (diameter 5 cm) placed in a sterile filtration apparatus (i) 70% ethanol, room temperature, 10 min; and then [(ii) UV radiation, room temperature, 30 min]; after the spontaneous filtration, the bacterial cells both in the filtered suspensions and in the obtained filtrates were quantified (Lencova et al., 2021a). Values of log_10_removal (CFU/ml) and retention rate (%) were calculated (Lencova et al., 2021b). At least three independent replicates were performed for each sample.

### Inhibition Assay for Functionalized PA Nanomaterials

#### Antibacterial Activity of PA Nanomaterials

Bacterial pure cultures in TSB were cultivated (24 h, 37°C); suspension ODs were adjusted to 1 McF and decimally diluted. A piece (1 cm×1 cm) of sterile PA was placed into the test tube containing 5 ml of bacterial suspension with an approx. concentration of 10^2^ CFU/ml. After the incubation (24 h, 37°C), CFU/ml were quantified. Bacterial suspensions without any added nanomaterial were used as controls. The log_10_suppression (CFU/ml) and the inhibitory rate (%) of PAs were calculated (Lencova et al., 2021b). At least three independent replicates were performed for each sample.

#### Biofilm Formation on PA Nanomaterials

One centimeter × one centimeter PAs were sterilized both after preparation and before each analysis by UV radiation (room temperature, 20 min). A sterile piece of nanomaterial was placed in a 12-well microtiter plate (Greiner Bio-One, Austria) containing 3 ml of single-bacteria suspension (TSB with an approx. bacterial cell concentration of 10^6^ CFU/ml). Microtiter plates were then cultivated (37°C, 48 h) for the formation of a single-species biofilm. Control biofilms were formed in a 12-well microtiter plate on polystyrene under the same conditions.

#### Determination of Viable Bacteria Forming Biofilm by CFU Enumeration

PAs with grown biofilms were washed five times with sterile distilled water and moved to a new, sterile microtiter plate; control biofilms formed in microtiter plates were washed five times with sterile distilled water. After drying (45 min, room temperature), 1 ml of MHB was added to the wells. Plates were sonicated (3 min, room temperature) in a sonication bath (Polsonic, Poland); MHB with released biofilm-forming cells was decimally diluted. Droplets of the dilution (20 μl) were plotted on Plate Count Agar (PCA, Merck, Germany) in triplicates and cultivated (24 h, 37°C). CFUs were counted, and values of biofilm formation [log_10_(CFU/ml)], biofilm formation suppression [log_10_suppresion (CFU/ml)], and biofilm formation rate (%) were calculated according to equations published in the previous study (Lencova et al., 2021a), and biofilm suppression (%) was calculated according to Eq. 1. At least three independent replicates were performed for each sample.

Equation 1: biofilm reduction calculation:


(1) Biofilm suppression[%]=100−biofilmformationrate

### Food Samples Assay

The assay protocols were designed according to Lin et al. (2018) and Göksen et al. (2021) and slightly modified. Fresh chicken breasts were purchased from a local market (Prague, Czechia), transferred to the laboratory within 10 min, and immediately analyzed. The chicken was cut into 10 g pieces, and the surface was sterilized with 70% ethanol (5 min). Half of the samples were left untreated [for total viable cells (TVC) enumeration], and half were immersed (15 min, room temperature) in the prepared *L. monocytogenes* CCM 7202 suspension with an approx. concentration of 10^7^ CFU/ml and dried (30 min, room temperature). All the samples were packed into sterilized (UV, 30 min) pieces (10 cm×10 cm) of aluminum foil (control), PA, PA/NAT, PA/RE, and PA/GTE, respectively (all procedures were performed aseptically in a laminar flow box). The packed chicken samples were stored separately at 7°C for up to 7 days. The below analyses were performed immediately (day 0) and after 1, 4, and 7 days of storage. All the samples were prepared and tested in at least triplicates for each analysis.

#### Antibacterial Activity of PA Nanomaterials Tested on Non-inoculated Chicken Breast (TVC Determination)

The non-inoculated stored fillets (10 g) were homogenized in a pulsifier (Microgen Bioproducts Ltd., Great Britain) for 1 min with 90 ml of sterile distilled water in a sterile blender bag (VWR, United States). Thus the filtrate representing the first decimal dilution of the sample was obtained. Another seven serial dilutions were prepared; droplets (20 μl) of all the dilutions were inoculated on PCA in triplicates. After cultivation (37°C, 48 h), the number of CFU/g was enumerated.

#### Antibacterial Activity of PA Nanomaterials Tested on Chicken Breast Inoculated With *Listeria monocytogenes* CCM 7202

The inoculated stored chicken samples (10 g) were homogenized and diluted as described above; droplets (20 μl) of all the dilutions were inoculated on Agar Listeria acc. to Ottaviani and Agosti (ALOA, Merck, Germany) in triplicates; *L. monocytogenes* forms blue-colored colonies with a transparent zone on ALOA agar. After cultivation (37°C, 24 h), the number of CFU/g was enumerated, and final *L. monocytogenes* CCM 7202 concentrations in the tested samples were determined.

#### pH Differences

The filtrate, the first sample dilution of both non-inoculated and inoculated samples was used for pH measurement with a calibrated pH meter (Radiometer Analytical, France). Each sample was measured in at least triplicate.

#### Sensory Analysis

Fresh chicken breast samples (non-inoculated) packed in aluminum foil, PA, PA/NAT, PA/RE, and PA/GTE were stored for 7 days at 7°C. Sensory evaluation of the samples (marked as A, B, C, D, and E for the purpose of a single-blind study) was performed on days 0, 1, 4, and 7 of storage. Appearance, color, texture, odor, and overall acceptance of the samples were evaluated by five panelists on a five-point scale (1 indicates ideal, specific properties for chicken breast; 5 marks unusual and unpleasant properties); the lower the final score, the better quality. The final acceptability score was enumerated as an average of partial results.

### Statistical Analysis

Statistical analysis was performed in program R (R Core Team, 2017) using the package vegan (Oksanen et al., 2016). All the experiments were performed in at least triplicates; the results are expressed as means and SDs. The normality of the data was established by the Shapiro–Wilk test; the data were considered normally distributed at *p* > 0.05. Multiple comparisons of the data were determined by one-way ANOVA, where the difference was assumed to be significant at *p* ≤ 0.05 and *p* ≤ 0.01.

## Results and Discussion

**Figure 1:**
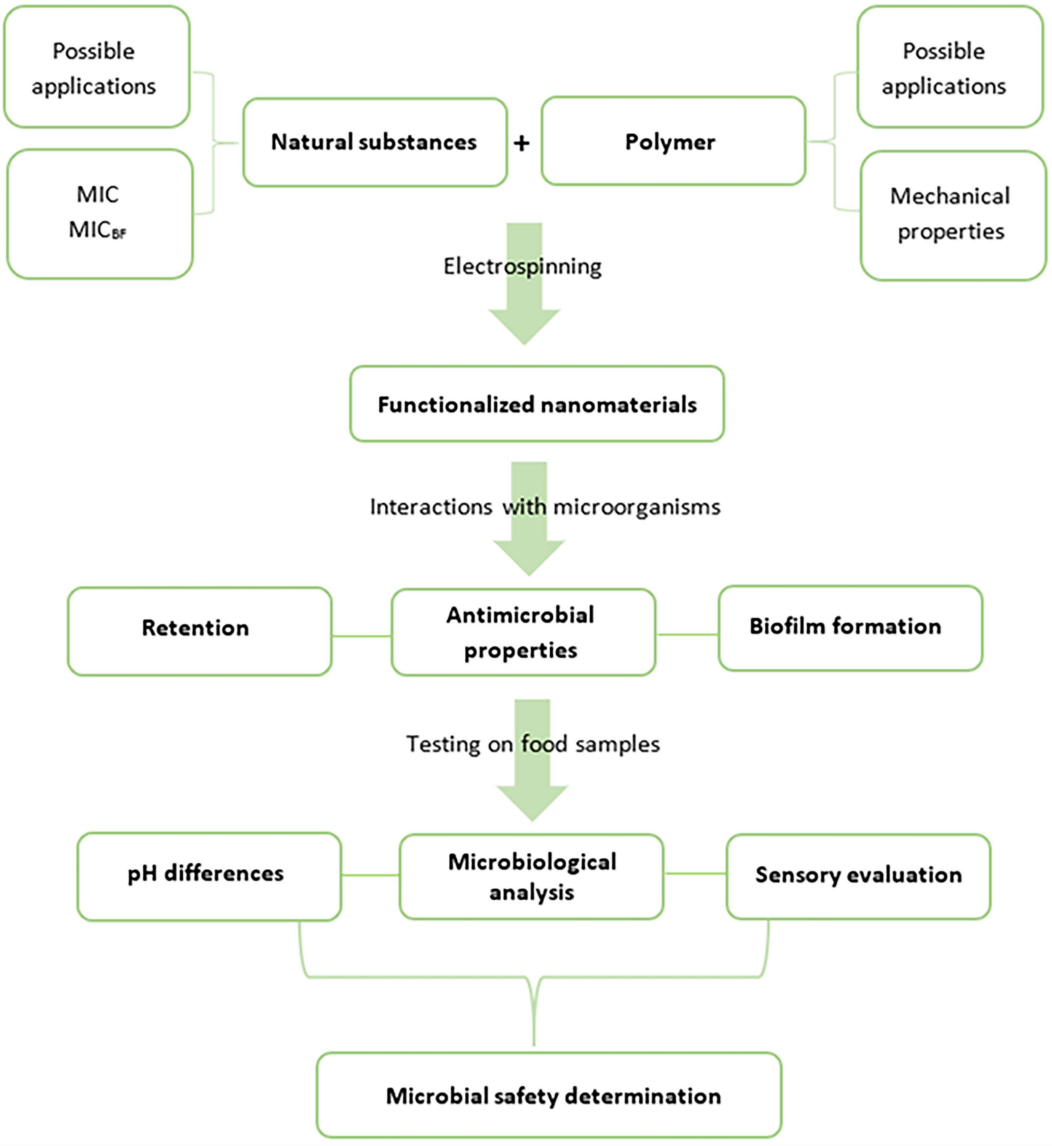
A schema of comprehensive development of microbiologically beneficial active food packaging (MIC, minimal inhibitory concentration and MIC_BF_, minimal inhibitory concentration for biofilm formation).

We present a unique and comprehensive design for the development of microbiologically safe nanomaterials that reduce the risks of foodborne infections and intoxications and can be used as food packaging or a packaging component (Figure 1). Studies covering all these aspects are rare (Babu et al., 2016), and usually, only address tests of the antimicrobial activities of the compounds used and corresponding functionalized nanomaterials (Nazari et al., 2019). Nevertheless, to be able to evaluate all the microbiological benefits of active food packaging, the following properties should also be considered (Figure 1): (i) the retention of undesirable microorganisms from the environment, that is, packaging acting as a barrier, (ii) evidence of antibacterial activity which prevents the growth of pathogens already present in the food or captured from the external environment, and (iii) inhibition of the biofilm formation of these pathogens. Simultaneously, all these properties of food packaging that positively affect the final microbiological safety of the product should not negatively affect its appearance, such as color, texture, odor, and overall acceptance.

### Antibacterial and Antibiofilm Effect of NAT, RE, and GTE

NAT, RE, and GTE were chosen as active natural substances suitable for PA’s functionalization. All these substances, known for their antibacterial activity against a wide spectrum of food pathogens (Campo et al., 2000; EFSA, 2009; Reygaert, 2014), are allowed for usage in the food industry (details of EU legislative is listed in Table 1). Their natural origin is an advantage for food applications, such substances are generally more acceptable for direct addition to food products than artificial preservatives (Topuz and Uyar, 2020).

**Table 1:** Natamycin, rosemary extract, and green tea extract EU legislation conditions.

Substance	Origin	EU food additive	EU legislative regulations	Approved applications	Max. dose
Natamycin	Produced by natural strains of *Streptomyces natalensis* or *Streptococcus lactis*	E 235	2015/647; 1333/2008 on food additives	Surface preservative for cheese products and dry and cured sausages	1 mg/dm^2^ in the outer 5 mm of the surface; 20 mg/kg (Nguyen Van Long et al., 2016)
Rosemary extract	Extraction of the leaves of the *Rosmarinus officinalis*	E 392	1333/2008 on food additives	Dehydrated milk; fats and oils; fruit and vegetable; nut butters; potato products; chewing gum; decorations, coatings, and fillings; meat products; fish and fishery products; eggs and eggs products; mustards; sauces; seasoning; potato-, cereal-, flour-, or starch-based snacks; and food supplements	15–400 mg/kg (EFSA Panel on Food Additives and Nutrient Sources added to Food et al., 2018)
Green tea extract	Extraction of the leaves of the *Camellia sinensis*	-	1334/2008 on flavorings and certain food ingredients with flavoring properties for use in and on foods; 1924/2006 on nutrition and health claims made on food (Wrona et al., 2017)	Active agent; can be used as a food additive in margarines, spreads, meat/seafood/poultry products	without limits (Regulation 1924/2006; Wrona et al., 2017)

**Figure 2:**
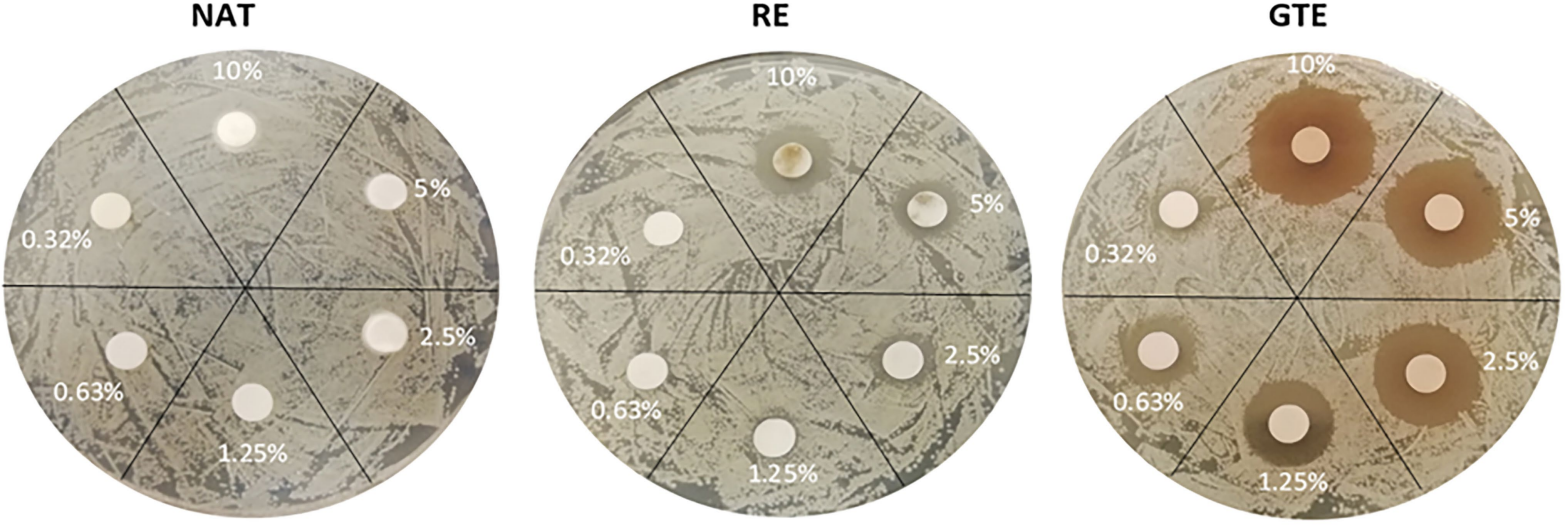
Influence of natural substances (NAT, RE, and GTE) in various concentrations (wt %) to viability of *Listeria monocytogenes* CCM 7202 (disk diffusion method results).

In our study, NAT, RE, and GTE effectively suppressed the growth and biofilm formation of all the tested bacterial strains. The MIC_dd_, MIC_md_, and MIC_BF_ values varied for substances and bacterial strains (Table 2; Figure 2; Supplementary Figure S1); however, at least one of the tested concentrations inhibited bacterial growth and biofilm formation. From the tested substances, the most effective was GTE, which exhibited the lowest MIC and MIC_BF_ values for all the bacterial strains with the highest inhibition effect for *L. monocytogene*s. Its antibacterial and antibiofilm effect is caused mainly by GTE catechins (polyphenols): (−)-epicatechin, (−)-epicatechin-3-gallate, (−)-epigallocatechin, and (−)-epigallocatechin-3-gallate (Reygaert, 2014). Epigallocatechin gallate is considered the main compound in green tea with inhibitory activity leading to the inhibition of bacterial growth and biofilm formation (Zhu et al., 2015). The mentioned catechins (i) damage the cell membrane (which leads to inhibition of the ability of the bacteria to bind to target cells, inhibition of biofilm-forming ability, and the inability of the bacteria to secrete toxins; Blanco et al., 2005; Sharma et al., 2012; Shin et al., 2012), (ii) inhibit fatty acid synthesis (*via* the inhibition of specific reductases FabG and FabI in bacterial type II fatty acid synthesis; Li et al., 2006), and (iii) inhibit enzyme activity (inhibit tyrosine phosphatase and cysteine proteinases in anaerobic bacteria, interfere with DNA replication by inhibition of DNA gyrase, and inhibit ATP synthase, reducing the ability of the bacteria to produce energy; Okamoto et al., 2003; Gradišar et al., 2007; Chinnam et al., 2010). They bind to the lipid bilayer in the cell membrane and may change the regulation of genes; the influence of GT catechins on upregulation/downregulation of 17 individual genes (with the major outcome of cell membrane damage) was confirmed in *E. coli* in the study of Cho et al. (2007). Further, GTE contains growth and biofilm-suppressing quorum sensing inhibitors (QSIs), which diminish protease activity and trimethylamine production (Zhu et al., 2015). Except these general antimicrobial mechanisms, GTE has an ability to evoke various other effects contributing to the antimicrobial effect in infected individuals, for example, inhibition of inflammation by increasing the synthesis of nitric oxide (Yamakuchi et al., 2008) and inhibition of angiotensin II and interleukin-6-induced C-reactive protein expression (Li et al., 2012).

**Table 2:** Determined MIC_dd_ (minimal inhibitory concentration determined with disc diffusion method), MIC_md_ (minimal inhibitory concentration determined with microdilution method), and MIC_BF_ (minimal inhibitory concentration for biofilm formation) of NAT, RE, and GTE for *Escherichia coli* CCM 4517, *L. monocytogenes* CCM 7202, *Salmonella enterica* CCM 7189, and *Staphylococcus aureus* CCM 3953.

	NAT (wt %)			RE (wt %)			GTE (wt %)		
	MIC_dd_	MIC_md_	MIC_BF_	MIC_dd_	MIC_md_	MIC_BF_	MIC_dd_	MIC_md_	MIC_BF_
*E. coli* CCM 4517	10.00	5.00	5.00	10.00	5.00	5.00	5.00	2.50	5.00
*L. monocytogenes* CCM 7202	10.00	1.25	1.25	1.25	2.50	2.50	0.32	1.25	0.63
*S. enterica* CCM 7189	10.00	2.50	2.50	5.00	5.00	5.00	5.00	2.50	2.50
*S. aureus* CCM 3953	10.00	2.50	2.50	10.00	2.50	5.00	5.00	2.50	2.50

The biological activity of RE is influenced mainly by phenolic compounds (stimulate antioxidant action), and content of compounds affecting microbial growth. Antimicrobial action is caused by 1,8-cineole, rosmarinic acid, rosmaridiphenol, carnosol, epirosmanol, carnosic acid, rosmanol, and isorosmanol. These compounds interact with the bacterial cell membrane, change genetic material, influence nutrients availability, alter electrons transport, cause leakage of cellular components, change fatty acids metabolism, and decrease the functionality of proteins (Nieto et al., 2018). The 1,8-cineole is recently considered to be the main one acting against pathogens (Göksen et al., 2021); rosmarinic acid further acts as a QSI and prevents biofilm formation (Galié et al., 2018). QSIs were recently proposed as a new generation of antimicrobial agents and are thus considered to be a new effective strategy for the eradication of food-associated bacterial biofilms (Gilles and Tom, 2015).

In comparison with GTE and RE, NAT is primarily effective against yeasts and fungi; it binds to the sterols (specifically to ergosterol) in their membranes, inhibits amino acid and glucose transport proteins, increases membranes permeability, and thus disrupts the cells, which leads to leakage and loss of cellular constituents (EFSA, 2009). Therefore, the lower suppression of bacteria, whose membranes lack sterols, in comparison with RE and GTE was expected. However, NAT was effective against the tested bacteria, and MIC/MIC_BF_ values were determined.

The overall results indicate that substances at concentrations from 1.25 to 2.5 wt% significantly inhibited bacterial growth; concentrations from 5.0 to 10.0 wt% suppressed cell growth completely. *Listeria monocytogenes* CCM 7202 was evaluated as the most sensitive strain, and *E. coli* CCM 4517 as the most resistant strain (Table 2). The tested Gram-positive bacteria were more sensitive to the substances used than the Gram-negative ones (*p* ≤ 0.05). The higher sensitivity of Gram-positive bacteria to antimicrobials is due to differences in their cell wall structure, thickness, and composition (Kramer et al., 2015). Gram-positive bacteria have a relatively thick cells wall (approx. 20–80 nm), while Gram-negative bacteria have a thin cell wall (approx. <10 nm). Next, the cell wall of Gram-positive bacteria is composed primarily of thick layer of peptidoglycan and contains teichoic acid, while the Gram-negative bacteria cell wall contains mainly a bilayer of phospholipids and only a thin layer of peptidoglycan. This all predetermines Gram-negative bacteria to be less sensitive to various antimicrobial substances (Kramer et al., 2015; Mai-Prochnow et al., 2016; Lencova et al., 2022). Given the current requirements for using the lowest amounts of additives in food and food contact materials, the functionalization of PA was done with NAT, RE, and GTE at a uniform concentration of 2.0 wt%. This concentration provides a good antibacterial and antibiofilm effect.

### Characteristics of Functionalized PA Nanomaterials

**Figure 3:**
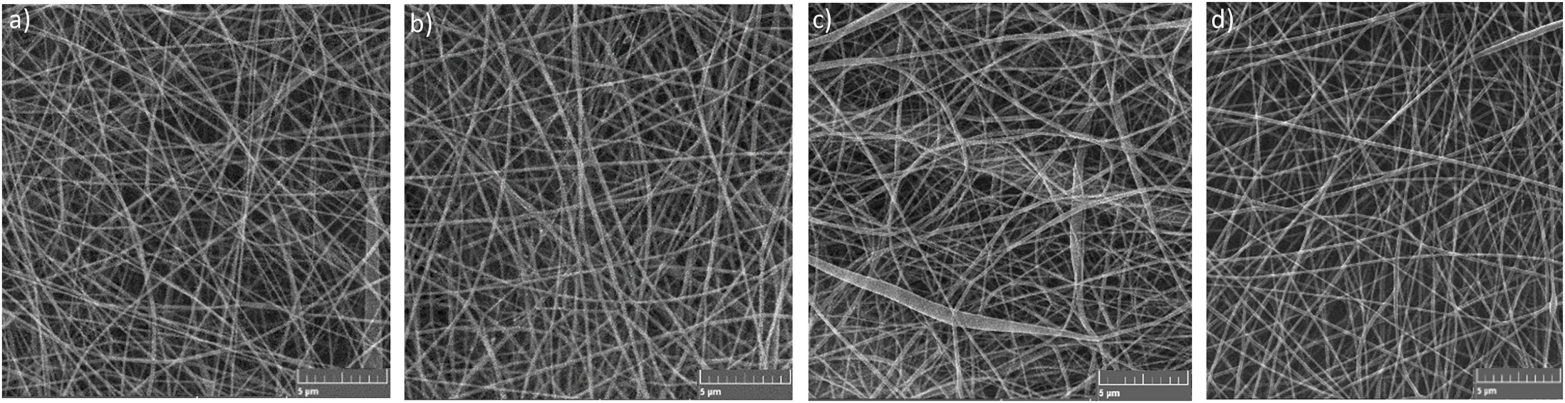
SEM images of **(a)** PA, **(b)** PA/NAT, **(c)** PA/RE, and **(d)** PA/GTE structures.

Four samples of electrospun PA nanomaterials were prepared *via* needleless electrospinning (NLES) and characterized in terms of their fiber diameter, surface density, and air permeability. NLES is one of the most used methods for nanofibrous materials preparation. This type differs from the classic electrospinning setup in the fabrication process—polymeric solution is electrospun directly from an open liquid surface. In comparison with conventional electrospinning, NLES overcome the problems with needle clogging, limited production capacity, and low yield (Partheniadis et al., 2020). The first NLES setup was patented and commercialized in the Czechia with the brand name Nanospider® (Jirsak et al., 2009; Partheniadis et al., 2020). As expected from our previous experiences with Nanospider® technology (Lencova et al., 2021a,b), all the prepared PAs exhibited good mechanical properties—were resistant, firm, and not prone to tearing. The prepared PAs were homogenous, no defects in continuous nanofibrous layers or significant variations in fiber diameters were recorded (Figure 3). PA is generally considered to be a suitable polymer for the production of homogenous and well-defined nanomaterials with precise and uniform characteristics (Matulevicius et al., 2014; Lencova et al., 2021a).

The non-functionalized PA contained no antimicrobial substance; the functionalized ones were electrospun from the polymer solution containing 2.0 wt% of NAT, RE, and GTE, respectively. During the production of Pas, all the food safety aspects were considered. The used substances (NAT, RE, and GTE) are allowed in food applications (Table 1) and the solvents (acetic acid, formic acid) are generally recognized as safe (GRAS) for use in foods and food packaging by Food and Drug Administration (FDA), respectively (FDA, 1980a,b). In case residues of the solvent system remain in the PA samples despite the evaporation step, they should not affect the safety of these nanomaterials. The influence of PA functionalization on their final properties was also evaluated. We did not notice any visible difference between morphology and structure of non-functionalized and functionalized PAs, thus can be assumed that addition of NAT, RE, and GTE into electrospun polymer solution did not influence the properties of the nanomaterials. The fiber diameter of PAs ranged from 80 to 140 nm. The average fiber diameters were the following: 103.6 ± 26.1 nm for PA, 108.2 ± 23.4 for PA/NAT, 105.7 ± 26.9 for PA/RE, and 106.8 ± 24.3 for PA/GTE. The surface density controlled by the spinning parameter was similar for all the PAs, 1.0 g/m^2^, and the air permeability ranged from 40 to 50 L/m^2^/s. The PAs antimicrobial properties are mainly affected by functionalization; however, their properties (fiber diameter and surface density) can also affect their bacterial colonization. The lower biofilm formation was observed on nanomaterials with low fiber diameter and low surface density (Lencova et al., 2021a). The given morphology was chosen to suppress biofilm formation and simultaneously ensure that the nanomaterials were not fragile and did not rupture during handling.

### Interactions Between PA Nanomaterials and Food Pathogens

#### Permeability Assay

The ability of the packaging materials to retain bacterial cells from the external environment is essential. By effectively retaining possible sources of food contamination, risks associated with food pathogens can be prevented. All the tested PAs provided excellent barrier properties (Table 3), and functionalization did not influence the overall retention ability (*p* ≥ 0.05). The log_10_removal ranged from 5.0 to 7.1 with a retention rate of 100.0%, with no significant difference between Gram-positive and Gram-negative bacteria (*p* ≥ 0.05).

**Table 3:** Retention of nanomaterials when filtering bacterial suspensions expressed as log_10_removal (CFU/ml) and %; antibacterial activity of nanomaterials relative to the control—bacterial suspension alone—and expressed as a bacterial growth suppression in log_10_(CFU/ml) and %; biofilm formation on nanomaterials related to the control—biofilm formation on polystyrene—expressed as biofilm formation suppression in log_10_(CFU/ml) and %.

	Bacterial strain	Bacterial cells retention/suppression	PA	PA/NAT	PA/RE	PA/GTE
PA’s retention of bacterial cells	*E. coli* CCM 4517	log_10_(CFU/ml)	7.0 ± 0.0	5.7 ± 0.9	7.0 ± 0.0	5.0 ± 1.0
		%	100.0 ± 0.0	100.0 ± 0.0	100.0 ± 0.0	100.0 ± 0.0
	*L. monocytogenes* CCM 7202	log_10_(CFU/ml)	5.2 ± 0.8	6.4 ± 0.8	5.0 ± 1.0	5.7 ± 0.8
		%	100.0 ± 0.0	100.0 ± 0.0	100.0 ± 0.0	100.0 ± 0.0
	*S. enterica* CCM 7189	log_10_(CFU/ml)	7.1 ± 0.8	5.6 ± 1.1	7.1 ± 0.0	7.1 ± 0.0
		%	100.0 ± 0.0	100.0 ± 0.0	100.0 ± 0.0	100.0 ± 0.0
	*S. aureus* CCM 3953	log_10_(CFU/ml)	6.5 ± 0.0	6.5 ± 0.0	6.5 ± 0.0	6.5 ± 0.0
		%	100.0 ± 0.0	100.0 ± 0.0	100.0 ± 0.0	100.0 ± 0.0
	Total average	log_10_(CFU/ml)	6.4 ± 0.8	6.0 ± 0.4	6.4 ± 0.8	6.1 ± 0.8
		%	100.0 ± 0.0	100.0 ± 0.0	100.0 ± 0.0	100.0 ± 0.0
Antibacterial activity of PAs (growth suppression)	*E. coli* CCM 4517	log_10_(CFU/ml)	0.1 ± 0.1	2.0 ± 0.4	1.7 ± 0.3	2.0 ± 0.1
		%	10.4 ± 15.7	98.5 ± 1.2	97.5 ± 1.5	99.0 ± 0.1
	*L. monocytogenes* CCM 7202	log_10_(CFU/ml)	0.2 ± 0.0	1.6 ± 0.7	1.8 ± 0.5	2.9 ± 0.4
		%	18.3 ± 13.4	92.4 ± 8.6	96.8 ± 2.4	99.9 ± 0.1
	*S. enterica* CCM 7189	log_10_(CFU/ml)	0.1 ± 0.1	2.5 ± 0.2	2.2 ± 0.6	2.8 ± 0.3
		%	11.0 ± 1.6	99.6 ± 0.2	98.6 ± 2.4	99.8 ± 0.1
	*S. aureus* CCM 3953	log_10_(CFU/ml)	0.1 ± 0.0	2.9 ± 0.4	3.0 ± 0.4	3.0 ± 0.5
		%	10.6 ± 4.8	99.8 ± 0.1	99.8 ± 0.1	99.8 ± 0.2
	Total average	log_10_(CFU/ml)	0.1 ± 0.0	2.3 ± 0.5	2.2 ± 0.5	2.7 ± 0.4
		%	12.6 ± 3.3	97.6 ± 3.0	98.2 ± 1.3	99.6 ± 0.3
Biofilm formation suppression on PAs	*E. coli* CCM 4517	log_10_(CFU/ml)	0.2 ± 0.1	2.2 ± 0.9	2.5 ± 0.1	2.7 ± 0.2
		%	25.3 ± 9.1	96.3 ± 4.3	99.5 ± 0.4	99.7 ± 0.2
	*L. monocytogenes* CCM 7202	log_10_(CFU/ml)	0.0 ± 0.0	1.7 ± 0.4	2.2 ± 0.4	2.9 ± 0.4
		%	4.6 ± 2.1	97.6 ± 1.2	98.9 ± 1.0	99.9 ± 0.1
	*S. enterica* CCM 7189	log_10_(CFU/ml)	0.1 ± 0.1	2.8 ± 0.2	2.5 ± 0.4	3.0 ± 0.4
		%	10.4 ± 7.3	99.8 ± 0.1	99.5 ± 0.3	99.9 ± 0.0
	*S. aureus* CCM 3953	log_10_(CFU/ml)	0.2 ± 0.1	2.6 ± 0.1	2.9 ± 0.4	2.7 ± 0.1
		%	21.5 ± 8.5	99.7 ± 0.2	99.7 ± 0.2	99.8 ± 0.1
	Total average	log_10_(CFU/ml)	0.1 ± 0.1	2.3 ± 0.4	2.5 ± 0.3	2.8 ± 0.1
		%	15.5 ± 8.3	98.4 ± 2.7	99.4 ± 0.6	99.8 ± 0.1

The permeability of nanomaterials (including permeability for microbial cells) depends directly on their morphology, especially on fiber diameter and surface density, which together determine the overall porosity and mechanical resistance (Lencova et al., 2021b). Based on the results, the prepared PAs were suitable for the efficient capture of bacterial cells (tested for the round-shaped 0.5–1.5 μm cells of *S. aureus* and rod-shaped 0.5–2 μm cells of *Enterobacteriaceae* and *L. monocytogenes*). Because the tested bacteria differed in morphology and their arrangement (staphylococcal cells typically clump into the shapes of grapes, whereas rod cells maintain individual or form clusters of non-specific shapes), PA retention is also expected for other microbial species.

Nanomaterials are usually tested as filtration membranes with inanimate particles, that is, dust particles (Matulevicius et al., 2014), and microbial suspensions are rarely used. For example, for staphylococci suspensions, electrospun PCL/Cloisite 30B membranes and PA nanomaterials were proven to be effective barriers (Babu et al., 2016; Lencova et al., 2021b). Thus, the results of this study extend current knowledge of the fundamental characteristic of PA nanomaterials, which is the first step toward food packaging that reduces microbial risks (Figure 1).

#### Inhibition Assay

##### Antibacterial Activity of PA Nanomaterials

Due to antibacterial activity, functionalized materials for direct food packaging should inhibit the growth of undesirable microorganisms present in the food or captured from the external environment. The antibacterial activity of prepared PAs was evaluated by a test in liquid medium using CFU enumeration based on which the growth inhibition was calculated. This test type was proven to be more conclusive than the standard one on agars with reading inhibition zones (Lencova et al., 2021b).

PA itself does not have any antibacterial activity (*p* ≥ 0.05), which is in accordance with our previous studies (Lencova et al., 2021a,b). In contrast, all the functionalized PAs reduced bacterial growth (1.7–3.0 log_10_suppression) with an inhibition rate from 92.4% to 99.9% (Table 3). The most pronounced effect was recorded for PA/GTE, reaching up to 3.0 log_10_supression and 99.9% bacterial cells reduction. Statistically, no significant difference was found between the overall effectivity of PA/NAT, PA/RE, and PA/GTE, nor between the inhibition of Gram-positive and Gram-negative strains by them (*p* ≥ 0.05).

Until now, polymers other than PA, for example, PCL (Veras et al., 2020) or polyvinyl alcohol (Göksen et al., 2021), have been used for functionalized materials with NAT, RE, and GTE. For example, Göksen et al. (2021) verified the antimicrobial effect of polyvinyl alcohol/RE nanofibers directly on food samples. Veras et al. (2020) determined the antifungal activity of PCL/NAT (2.5–8.2 μg of NAT per 1 mg of PCL) first on inoculated agar plates and then on soft cheese samples; the low toxicity of PCL/NAT nanofibers was confirmed and they were evaluated as a promising strategy for the control of microbiological safety in food systems.

Various electrospun polymers and active substances have been successfully used for food packaging (Lin et al., 2018; Pan et al., 2019). However, only a few studies have addressed their direct antimicrobial effect. Lin et al. (2018) prepared highly effective ε-polylysine/chitosan nanofibers against *S. enterica* and *S. tymphimurium*, and Pan et al. (2019) confirmed the high antibacterial effect of polyvinyl alcohol/β-cyclodextrin nanofibers against *E. coli* and *S. aureus*. Both studies used the disc diffusion method for their antimicrobial tests. The log reduction method was used by Nostro et al. (2012), who tested the effect of carvacrol (7 wt%) and cinnamaldehyde (7 wt%) in polymeric nanocomposites. Reductions from 1 to 4 log CFU of *E. coli*, *S. aureus*, *S. epidermidis*, and *L. monocytogenes* were determined. The outcome of the published studies is that diverse nanofibrous structures may effectively transport and control the release of antimicrobial substances and thus be a promising strategy for reducing the amounts of drugs, resulting in a positive effect on human health and the environment (Lopes and Brandelli, 2018; Veras et al., 2020).

##### Antibiofilm Activity of PA Nanomaterials

Biofilms in the food industry are one of the main threats to consumers health, especially for pathogenic bacteria, such as *E. coli*, *L. monocytogenes*, *Salmonella* spp., and *S. aureus*. These bacteria easily form biofilms on various surfaces, including food packaging. Due to the strong antibacterial effect of functionalized PAs toward these microorganisms, the next step (Figure 1) was to verify their biofilm suppression (Table 3). The non-functionalized PA slightly suppressed biofilm formation (*p* ≤ 0.05) compared to the control (polystyrene). Thus, neat PA nanofibers do not enhance biofilm formation and do not increase the risks associated with biofilm formation compared to conventional materials. Functionalized PAs, when compared with both control and neat PA, significantly (*p* ≤ 0.01) suppressed biofilm formation (1.7–3.0 log_10_CFU/ml) with the inhibition rate ranging from 96.3% to 99.9%. Statistically, no difference was determined between the antibiofilm activity of PA/NAT, PA/RE, and PA/GTE (*p* ≥ 0.05). All of these natural origin preservatives are suitable antibiofilm agents for polymer functionalization.

Babu et al. (2016) claimed that the antibiofilm activity of the material is a crucial property determining its microbial safety and is one of the major challenges in the food industry (Naskar et al., 2018). Despite this, studies of the anti-biofilm properties of food packaging materials are rare. Babu et al. (2016) prepared PCL/cloisite 30B thin films, that exhibited significant antibiofilm activity against S. haemolyticus and *S. epidermidis*. Their antibiofilm effect was tested in terms of the suppression of biofilm formation in the surroundings of the nanocomposites. Crystal violet (CV) staining was used for quantifying the biofilm formed in wells of a microtiter plate, in which both bacterial suspension and a nanocomposite were present. Naskar et al. (2018) used the same CV approach for confirming the antibiofilm activity of a polyethylene glycol-capped Ag–ZnO–graphene nanocomposite against *S. aureus* and *P. aeruginosa*. Nostro et al. (2012) studied and demonstrated the antibiofilm activity (up to 90% effectivity) of carvacrol and cinnamaldehyde (3.5–7 wt%) polymeric films based on the same principle as we used in this paper—in terms of lowering biofilm biomass formed on the material’s surface.

Due to the frequent and rapid formation of biofilms in food industry environments (Galié et al., 2018), we recommend including antibiofilm activity determination in the standard testing procedure (Figure 1). We believe that these unwanted accumulations of pathogens could be limited or entirely suppressed by appropriate functionalization when higher concentrations of active substances are used.

### Food Samples Assay

**Figure 4:**
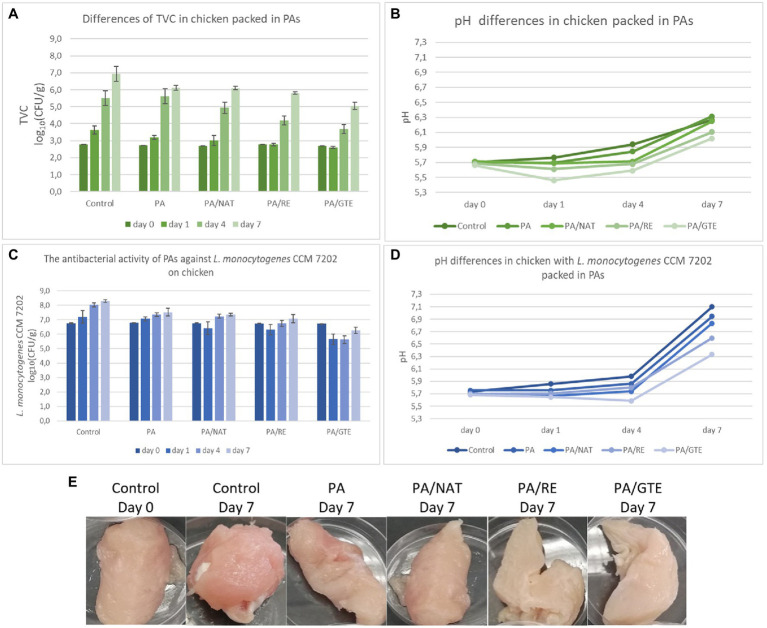
The antimicrobial activity of PAs against *L. monocytogenes* CCM 7202 on chicken samples **(A)** and differences in pH **(B)** after 7 days storage at 7°C; the influence of PAs on total viable count (TVC) of microorganisms in non-inoculated chicken samples **(C)**, differences in pH **(D)**, and appearance change **(E)** after 7 days storage at 7°C.

To appropriately evaluate the advantages of using nanomaterials, their effectiveness should be confirmed not only with model, but also under real conditions (Figure 1). The effect of functionalized PAs on prolonging the shelf life of chicken breast samples and their antimicrobial activity against *L. monocytogenes* CCM 7202 on inoculated chicken breast samples was evaluated for 7 days at 7°C (Figure 4). *Listeria monocytogenes* was selected as a model bacteria for this assay because it’s transmission from reservoirs to the poultry meat is common and as well its presence in poultry meat has been monitored with increasing occurrence during last years—there is nothing during the poultry processing that precludes *Listeria* spp. survival nor persistence on poultry meat (Rothrock et al., 2017). Further, it dispones the ability to grow under low temperatures, which makes it the most suitable from the tested bacteria to monitor the effect of PAs on bacterial growth during set storage conditions. The storage temperature of 7°C was chosen as a value corresponding to the average temperature in a refrigerator. The studies testing the effect of packaging were done at various temperatures from 4°C to 25°C, usually on chicken breast samples inoculated with *L. monocytogenes* or *Salmonella* spp. (Lin et al., 2018; Göksen et al., 2021); tests with non-inoculated food samples are rare (Pan et al., 2019).

PA itself reduced both TVC and *L. monocytogenes* CCM 7202 by 0.8 log_10_(CFU/g) after 7 days of storage, which means that PA decreased the growth of undesirable microorganisms and protected the food samples better than aluminum foil. The nanostructured materials themselves could reduce cell proliferation (Abrigo et al., 2015; Lencova et al., 2021a) and enhance food protection, which can be further improved by the functionalization of nanomaterials. All the prepared PAs containing 2.0 wt% of the active substance significantly suppressed both TVC in non-inoculated and *L. monocytogenes* CCM 7202 in inoculated samples [PA/NAT (*p* ≤ 0.05), PA/RE (*p* ≤ 0.05), and PA/GTE (*p* ≤ 0.01)]. The highest effect was detected for the nanomaterial PA/GTE, which decreased TVC by 1.9 log_10_(CFU/g) (equivalent to a 98.9% reduction of bacterial cells) and *L. monocytogenes* by 2.1 log_10_(CFU/g) (equivalent to a 99.0% reduction of bacterial cells).

Further, the overall sample appearance was monitored. During storage, the pH of control samples increased from 5.7 to 6.3 and to 7.1 for non-inoculated and inoculated samples, respectively. The increase in pH was generally smaller for the meat packed in various PAs (Figure 4), with the lowest change detected for PA/GTE (*p* ≤ 0.05 from the control). The differences in pH values are influenced by microbial growth, oxidation, or proteolytic activity (Lin et al., 2018; Göksen et al., 2021). Chicken breast fillets are rich in proteins and amino acids, and a pH increase in such samples is considered primarily to be an index of an increasing number of spoilage microorganisms (Göksen et al., 2021). PA nanomaterial packaging itself slowed down this spoilage, and functionalization, especially by GTE, increased this effect.

Sensory analysis (Tables 4 and 5) was performed for non-inoculated chicken breast samples packed in aluminum foil (controls) and various PAs. No adverse effect of PAs on appearance, texture, odor, or overall acceptance was recorded. PA itself maintained the sensory quality of the samples longer than controls. The meat samples packed in functionalized PAs remained at acceptable quality longer than both controls and neat PA; mainly, the texture and odor were influenced positively. The best sensory properties after 7 days of storage were determined for samples packed in PA/GTE.

**Table 4:** Sensory evaluation of chicken breast fillets samples packed in aluminum foil (control), PA, PA/NAT, PA/RE, and PA/GTE and stored for 7 days at 7°C.

Packaging material	Sensory parameter				
	Appearance	Color	Texture	Odor	Overall acceptance
Control	4.8 ± 0.4	4.8 ± 0.4	4.3 ± 0.4	5.0 ± 0.0	5.0 ± 0.0
PA	3.8 ± 0.4	3.3 ± 0.8	3.8 ± 0.8	4.5 ± 0.5	4.3 ± 0.8
PA/NAT	3.3 ± 0.4	3.0 ± 1.0	3.5 ± 0.5^*^	3.5 ± 0.5	3.8 ± 0.8
PA/RE	3.0 ± 0.0	2.5 ± 0.5^*^	3.5 ± 0.5^*^	3.3 ± 0.4	3.3 ± 0.4
PA/GTE	2.8 ± 0.4^*^	2.5 ± 0.5^*^	3.5 ± 0.5^*^	3.0 ± 0.7^*^	2.8 ± 0.4^*^

In the day 0, all the values were 1.0 ± 0.0 (indicating optimal quality of the samples).

* Marks the best results in a column.

**Table 5:** Sensory evaluation of chicken breast fillets samples packed in aluminum foil (control), PA, PA/NAT, PA/RE, and PA/GTE determined as final score of acceptability during 7 days storage at 7°C (values 1.0 ± 0.0 indicate the optimal quality).

Packaging material	Day of storage			
	Day 0	Day 1	Day 4	Day 7
Control	1.0 ± 0.0^*^	1.5 ± 0.2	2.3 ± 0.4	4.8 ± 0.3
PA	1.0 ± 0.0^*^	1.5 ± 0.2	2.1 ± 0.2	3.9 ± 0.4
PA/NAT	1.0 ± 0.0^*^	1.4 ± 0.1^*^	2.2 ± 0.3	3.4 ± 0.3
PA/RE	1.0 ± 0.0^*^	1.4 ± 0.1^*^	1.6 ± 0.1^*^	3.1 ± 0.3
PA/GTE	1.0 ± 0.0^*^	1.4 ± 0.1^*^	1.7 ± 0.1^*^	2.9 ± 0.3^*^

* Marks the best results in a column.

Many studies discuss the effect of functionalized (nano)materials on prolonging the shelf life of food samples. However, these have been predominantly done on materials without nanostructure. For example, NAT was used as an active substance in chitosan films for prolonging the storage life of cheese (Fajardo et al., 2010), or in polylactic acid films protecting semi-soft cheese against fungi and yeasts (Lantano et al., 2014). In connection with nanofibers, Veras et al. (2020) prepared PCL/NAT that prolonged cheese shelf life. RE and GTE were also added into various matrices for food packaging improvements, but again functionalized nanofibers were tested sporadically. RE has been incorporated into starch films as potential food packaging (Piñeros-Hernandez et al., 2017) and into polyvinyl alcohol nanofibers used for chicken breast fillets inoculated with *L. monocytogenes* (Göksen et al., 2021). In comparison with controls, RE polyvinyl alcohol nanofibers suppressed *L. monocytogenes* growth for approx. 2 log_10_(CFU/g) and also slightly decreased the pH of samples after 7 days storage at 4°C. Ethylene vinyl alcohol copolymer modified with GTE exhibited significant antioxidant activity and was evaluated as a suitable packaging for various types of foods (López de Dicastillo et al., 2011). The other example is GTE chitosan-coated plastic films, which reduced *L. monocytogenes* counts (2.65–3.2 log CFU/cm^2^) in ham steaks during room temperature storage (Vodnar, 2012). Recently, the antioxidant effect of GTE-modified PAs was confirmed on minced meat samples (Borzi et al., 2019); microbiological quality testing was not included. Until now, GTE nanofibrous materials were developed mainly for medical applications (Sadri et al., 2015), and their suitability for food packaging has not been studied.

In summary, this study confirms the potential of NAT, RE, GTE, and functionalized PA nanofibers as suitable materials for food packaging applications in terms of their microbiological safety and even benefits. All the functionalized PAs showed potential at lowering microbial risks resulting from food contamination, with PA/GTE being the most effective one. Furthermore, a comprehensive methodology (Figure 1) for evaluating the nanofibrous active food packaging for prolonging the shelf life was designed for the first time, and all the steps were verified.

Presented results are a good basis for a selection of appropriate nanomaterials applicable in functional food packaging. Nevertheless, a number of areas and tasks remain to be addressed before their final application. It would be appropriate to test PAs on other food products prone to microbial spoilage and their long-term stability. For our analysis, we selected four important food pathogens contaminating a variety of foods, but microbiological analysis can be extended to other pathogens or groups of spoiling microbiota (e.g., specifically in case of chicken meat, to *Campylobacter* spp. and psychrotrophic bacteria monitoring). From the chemical view of chicken meat quality, total volatile basic nitrogen (TVB-N) as a biomarker of the degradation of proteins and amines in meat (Bekhit et al., 2021) and peroxide value (PV) and thiobarbituric acid reactive substances (TBARS) as markers of lipid peroxidation (Song and Shurson, 2013) could be further evaluated. The final part should be the verification of non-toxicity and determination of mechanical properties of the final packaging made from the presented PAs or containing the PAs in packaging structure as an active part.

Also, further possibilities and future challenges open up for the development of functional materials, based on the presented scheme of microbiological analysis (Figure 1). For example: (i) higher concentrations of the substances may be used for nanomaterials functionalization, (ii) combinations of various natural substances with a possible synergistic effect may be tested this way, or (iii) nanomaterials with optimally adjusted morphology could provide even better results. In addition, not only single-species biofilms but also mixed-species biofilms pose a current threat in the food industry (Yuan et al., 2020). In the near future, it will be necessary to test the antibiofilm activity of the new packaging (nano)materials against them as well.

## Conclusion

Nanofibers have considerable potential in the development of active food packaging, maintaining food quality and safety. By functionalizing nanomaterials with natural substances, a prolonged food shelf life can be achieved without the need for preservatives. Electrospun PA nanofibers functionalized with NAT, RE, and GTE were prepared and compared for the first time. The nanomaterials were tested according to a comprehensive methodology for food packaging development, which could provide microbiological benefits and enhance food shelf life and microbial safety. The antibacterial effect of NAT, RE, and GTE for common food pathogens (*E. coli*, *L. monocytogenes*, *S. enterica*, and *S. aureus*) was verified, and MIC and MIC_BF_ values were determined. The prepared PA/NAT, PA/RE, and especially PA/GTE were proven to be effective at bacteria retention, growth inhibition, biofilm formation suppression, and at prolonging chicken breast shelf life. These benefits can lead to a reduction in risks associated with foodborne infections and intoxications. We believe that such nanomaterials can be used as food packaging/packaging components and should be studied further. Finally, in the future, they could be used as an effective and ecological alternative to standard packaging.

## Data Availability Statement

The original contributions presented in the study are included in the article/Supplementary Material, further inquiries can be directed to the corresponding author.

## Author Contributions

SL: conceptualization, methodology, investigation, visualization, data curation, and writing—original draft and revised manuscript preparation. HS and KZ: data curation, formal analysis, and supervision. MM: methodology, investigation, and resources. KD: formal analysis, resources, and supervision. All authors contributed to the article and approved the submitted version.

## Funding

The study was supported by the grants of Specific University Research—grant no. A2_FPBT_2021_001 and grant no. A1_FPBT_2021_005, funding the Methodology Development and Microbiological Analyses, and from the grant OP PIK CZ.01.1.02/0.0/0.0/16_084/0009936, within which nanofibers were prepared.

## Conflict of Interest

MM is employed by NanoMedical, Liberec, Czechia.

The remaining authors declare that the research was conducted in the absence of any commercial or financial relationships that could be construed as a potential conflict of interest.

## Publisher’s Note

All claims expressed in this article are solely those of the authors and do not necessarily represent those of their affiliated organizations, or those of the publisher, the editors and the reviewers. Any product that may be evaluated in this article, or claim that may be made by its manufacturer, is not guaranteed or endorsed by the publisher.

## Supplementary Material

Supplementary Figure S1 Influence of natural substances (NAT, RE, and GTE) to viability of bacterial cells [*E. coli* CCM 4517 (EC), *L. monocytogenes* CCM 7202 (LM), *S. aureus* CCM 3953 (SA), *S. enterica* CCM 7189 (SALM)]; **(A)** spectrophotometric measurement of bacterial cells viability, **(B)** Influence of natural substances (NAT, RE, and GTE) on biofilm formation.
