# Supplementary figures and images for: Potential of Polyamide Nanofibers With Natamycin, Rosemary Extract, and Green Tea Extract in Active Food Packaging Development: Interactions With Food Pathogens and Assessment of Microbial Risks Elimination
Source: Front Microbiol. 2022 Mar 15;13:857423. doi: 10.3389/fmicb.2022.857423 (PMC8965076; doi:10.3389/fmicb.2022.857423)

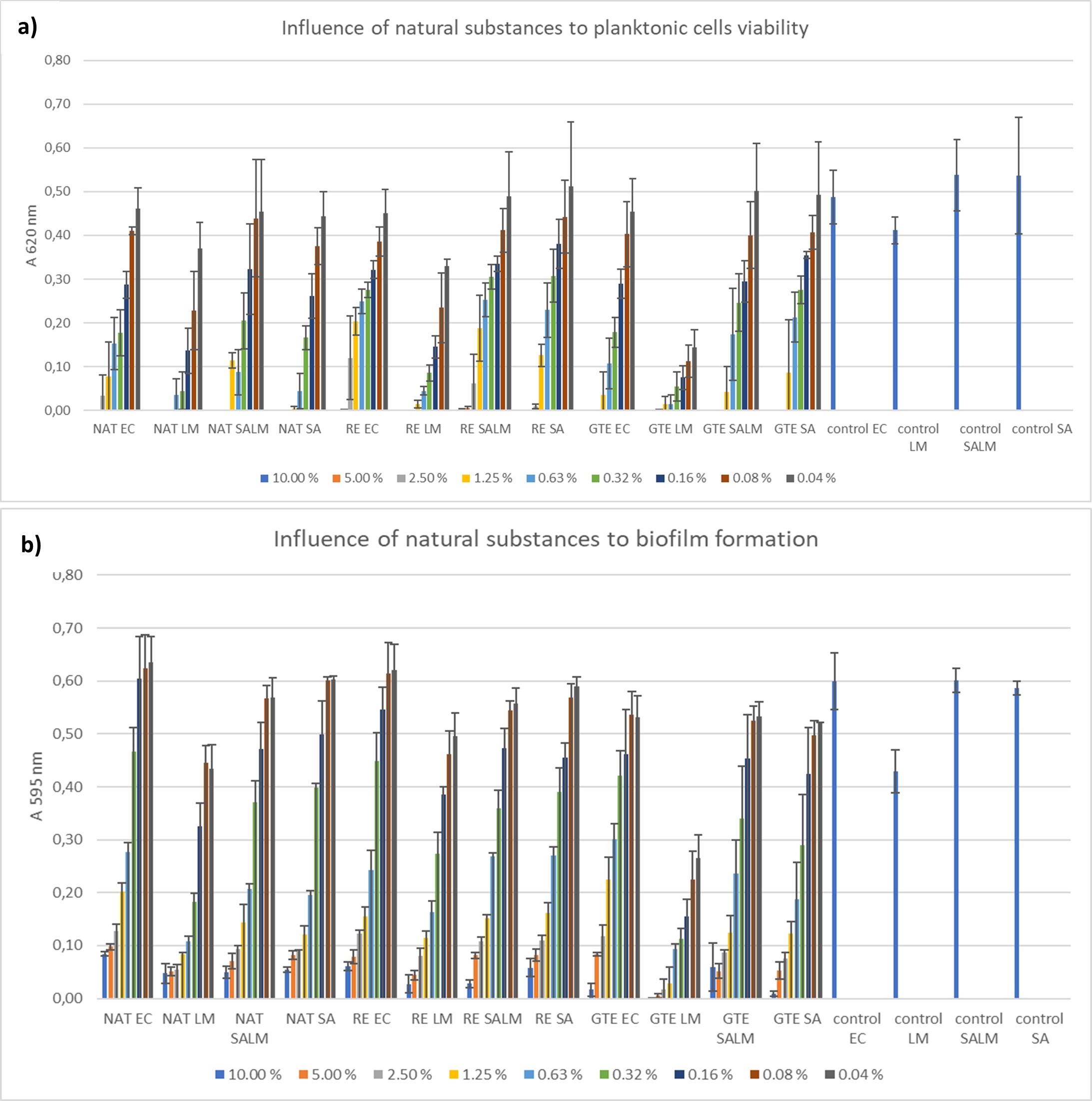

Supplement: Supplementary Figure S1 — Influence of natural substances (NAT, RE, and GTE) to viability of bacterial cells [E. coli CCM 4517 (EC), L. monocytogenes CCM 7202 (LM), S. aureus CCM 3953 (SA), S. enterica CCM 7189 (SALM)]; (A) spectrophotometric measurement of bacterial cells viability, (B) Influence of natural substances (NAT, RE, and GTE) on biofilm formation. [file Image_1.JPEG]
